# The alien ascidian *Styela clava* now invading the Sea of Marmara (Tunicata: Ascidiacea)

**DOI:** 10.3897/zookeys.563.6836

**Published:** 2016-02-15

**Authors:** Melih Ertan Çinar

**Affiliations:** 1 Ege University, Faculty of Fisheries, Department of Hydrobiology, 35100, Bornova, İzmir, Turkey

**Keywords:** Invasive alien species, *Styela clava*, Ascidiacea, Tunicata, Sea of Marmara

## Abstract

During the implementation of a large project aimed to investigate the benthic community structures of the Sea of Marmara, specimens of the invasive ascidian species *Styela clava* were collected on natural substrata (rocks) at 10 m depth at one locality (Karamürsel) in İzmit Bay. The specimens were mature, containing gametes, indicating that the species had become established in the area. The Sea of Marmara seems to provide suitable conditions for this species to survive and form proliferating populations.

## Introduction

The Sea of Marmara is unique in having two stratified water layers separated by a halocline, generally developing at 20-25 m depths (Besiktepe et al. 1994). The upper layer is composed of brackish water originating from the Black Sea, while, the lower layer comprises marine water from the Aegean Sea. This sea has been under great anthropogenic pressures, mainly due to crowded cities situated along its coastlines (including İstanbul), and the presence of many industrialized regions, in particular, İzmit Bay. Pollution from different sources has caused hyper eutrophication (Aral 1992) and occasionally anoxia (Basturk et al. 1990) in some areas. Moreover, the establishment of some invasive alien species [e.g. the ctenophore *Mnemiopsis leidyi* Agassiz, 1865, the asteroid *Asterias rubens* Linnaeus, 1758 and the gastropod *Rapana venosa* (Valenciennes, 1846)] in the basin has made conditions worse (Çinar et al. 2011). The mussel and oyster beds in the sea have been largely destroyed by the aforementioned asteroid and gastropod.

The invasive alien species are known to have great impacts on native communities and often make complete changes to ecosystems that cannot be rectified (Ruiz et al. 1997). The eastern Mediterranean Sea is one of the known regions that hosts high numbers of alien species, due to its proximity to the Suez Canal and high rate of maritime traffic (Çinar et al. 2012). This region includes 75% of the total number of alien species reported for the whole of the Mediterranean Sea (Zenetos et al. 2012). Seventeen alien ascidian species were reported from the Mediterranean Sea (see Zenetos et al. 2012), some of which, such as *Distaplia bermudensis* Van Name, 1902, *Microcosmus squamiger* Michaelsen, 1927 *Botrylloides violaceus* Oka, 1927 and *Didemnum vexillum* Kott, 2002, have become invasive in some areas, especially in the western Mediterranean and Adriatic Sea (Mastrototaro and Brunetti 2006; Occhipinti-Ambrogi 2000; Turon et al. 2007; Tagliapietra et al. 2012). The lessepsian invaders such as *Symplegma brakenhielmi* (Michaelsen, 1904), *Herdmania momus* (Savigny, 1816), *Microcosmus exasperatus* Heller, 1878 and *Phallusia nigra* Savigny, 1816 densely colonize both natural habitats and man-made structures in coastal regions of the eastern Mediterranean (in Levantine Sea), gradually extending their distributions to the north and west, including the Aegean Sea (Çinar et al. 2006; Kondilatos et al. 2010; Thessalou-Legaki 2012; Evans et al. 2013; Ramos-Esplá et al. 2013). Shenkar and Loya (2009) reported 7 alien species along the Mediterranean coast of Israel, including *Microcosmus exasperatus* Heller, 1878. A total of 4 alien ascidian species [*Phallusia nigra*, *Herdmania momus*, *Microcosmus exasperatus* and *Symplegma brakenhielmi*] have been reported along the Levantine and Aegean coasts of Turkey up to date, but no alien ascidian species have been encountered in the Sea of Marmara and the Black Sea coasts of Turkey (Çinar 2014).

During a TUBITAK project (number 111Y268), specimens of *Styela clava* Herdman, 1881 were encountered and photographed in one locality, Karamürsel, located in Izmit Bay. This sessile and solitary ascidian species is native to the north-western Pacific but now occurs worldwide, due to anthropogenic transport (Carlisle 1954; Millar 1960; Holmes 1976; Christiansen and Thomsen 1981; Berman et al. 1992; Cohen et al. 1998; Lambert and Lambert 1998; Minchin et al. 2006; Davis and Davis 2007; Ross et al. 2007; Hayward and Morley 2009). It is mainly characterized by its tunic shape and long stalk. This species was first reported in the Mediterranean Sea in June 2005, in Bassin de Thau (France) and was thought to have been transferred to the area by shellfish transfer (Davis and Davis 2008). This species was also recorded in the Black Sea in a species list of the macro-zoobenthos associated with a mussel facies inside the Constanta Sud-Agigea Seaport situated on the coast of Romania (Micu and Micu 2004). The species generally colonizes areas of shallow water and is especially abundant 10-200 cm below the sea surface, occasionally inhabiting hard substrate at depths of 15-40 m (Lützen 1999). However, Kott (2008) found it at 100 m depth in Shark Bay (Western Australia).

The aim of this paper is to report this species in the Sea of Marmara and to give additional information regarding its morphological and ecological characteristics.

## Material and methods

**Figure 1.**
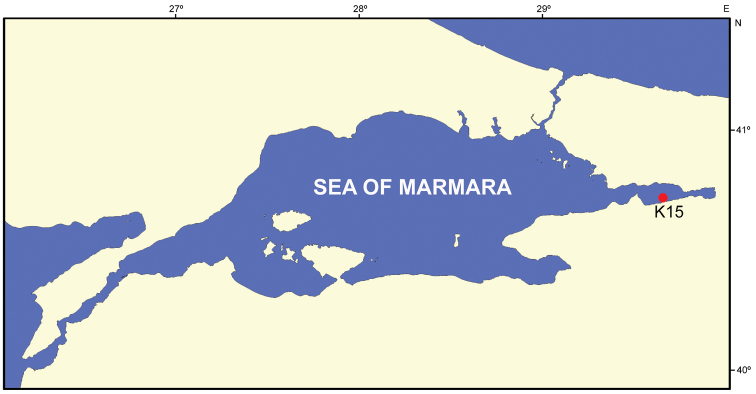
Map of the sampling site.

Specimens of *Styela clava* were collected at one locality (Karamürsel, K15, İzmit Bay, 40°41'38"N-29°36'26"E) in the Sea of Marmara at 10 m depth on rocks via scuba-diving on 01 October 2012 (Figure 1). The animals were randomly sampled and fixed with 4% formaldehyde in the field. In the laboratory, specimens were rinsed with tap water and preserved in 70% ethanol. Specimens were deposited at the Museum of Faculty of Fisheries, Ege University (ESFM).

## Results and discussion

### The description of *Styela clava*

**Figure 2.**
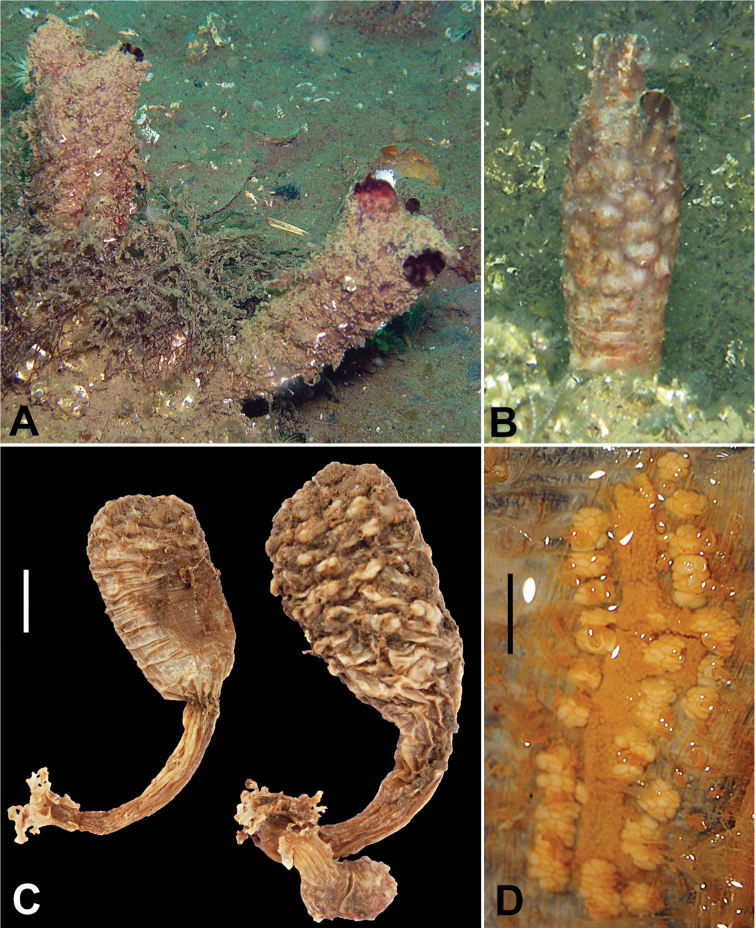
*Styela clava*, **A** Live animals densely covered with sediment and epibionts at K15 **B** Live animal almost bare at same station **C** Fixed specimens **D** Gonads. Scale bars: **C** = 1 cm, **D** = 2 mm.

Four specimens (registration code: ESFM-TUN/2012-1) were collected in the Sea of Marmara from station K15 at 10 m depth on rocks. Specimens are stalked and sessile. The body is more or less cylindrical, tapering to stalk. The body of the largest specimen is 5.5 cm long and 2 cm wide. The smallest specimen is 3.2 cm long and 1.8 mm wide. The specimen stalk reaches 3.5 cm long (Figure 2A–C). The siphons are short and placed anteriorly; the branchial siphon is more obvious than the atrial siphon. The external body surface is leathery, wrinkled, with irregular rounded conical warts (Figure 2B, C). The body color is white in fixed specimens (Figure 2C), but is chocolate-brown when alive (Figure 2A, B). Apertures have alternate longitudinal pale brown and dark brown stripes (Figure 2A).

The branchial tentacles are simple. There are four branchial folds curved inwards on each side of the posterior part of the body. The branchial sac has numerous rows of straight stigmata. The gut is placed on the left side of the branchial sac, like a simple vertical loop. Gonads are long, parallel to each other, consisting of a central ovarian tube with testis follicles on the body wall along the each side of the ovary (Figure 2D). In the largest specimen, gonads are placed on both sides (2 on the left side and 4 on the right side), consisting of a long ovary surrounded by male follicles (Figure 2D).

### The epibionts of *Styela clava*

The specimens of *Styela clava* from the Sea of Marmara were generally covered by sediment and some epibionts, such as *Diadumene cincta* Stephenson, 1925, *Spirobranchus triqueter* (Linnaeus, 1758) and green algae. The former species is known also to be an alien species, probably transferred to the area by shipping from the north-east Atlantic (Çinar et al. 2014). Lützen (1999) reported various epibionts on *Styela clava* in the North Sea, from tufts of red or green algae to ascidians including smaller specimens of the same species as well as *Ascidiella aspersa* (Müller, 1776) and *Botryllus schlosseri* (Pallas, 1766).

### The density of *Styela clava*

During many scuba dives and snorkeling trips performed along the Sea of Marmara in September and October 2012 (30 stations), this species was only encountered at station K15 (İzmit Bay, Karamürsel) and only 10 specimens were observed at a depth of 10 m on natural habitats (on rocks). The density of the species was approximately 1 ind.m^-2^. The dominant macrozoobenthic species sharing the same habitat with *Styela clava* were *Mytilus galloprovincialis* Lamarck, 1819, *Spirobranchus triqueter*, *Diadumene cincta*, *Rapana venosa* and *Asterias rubens*. The latter three species are also invasive alien species in the Sea of Marmara. *Styela clava* has been known to become extremely dominant in some areas, attaining a density of 1000 ind.m^-2^ in European waters (Lützen 1999; Minchin et al. 2006). Micu and Micu (2004) reported a density of 4 ind.m^-2^ and biomass (dry-weight) of 22.8 g.m^-2^ in the Agigea seaport in the Black Sea (Romanian coast).

### The survival requirements of *Styela clava*

This species has a club-shaped body that can reach a length of 200 mm and attaches to hard substrata by an expanded membranous plate (Minchin et al. 2006). It reaches maturity at a size of 5 to 7.5 cm after ten months of settlement (Davis and Davis 2007). It is a hermaphroditic species and has a pelagic lecithotrophic larva that rarely travels more than a few centimeters according to Davis et al. (2007), but probably travels much father due to currents. It is known to tolerate temperatures ranging from -2 to +23 °C and salinity from 20 to 32 psu (Davis and Davis 2007). At this particular station within the Sea of Marmara, the temperature was near the maximum known tolerance limit of the species (22.6 °C) and the salinity was 23 psu. Similar environmental conditions were also encountered at different sampling stations in the Sea of Marmara, but no animals of this species were found at those stations. These findings might suggest that the species was found at the area of first establishment. In the Sea of Marmara, the summer surface water temperatures and salinity at the sampling site (İzmit Bay) were reported to be 25 °C and 23 psu, respectively (Isinibilir et al. 2008). Winter surface water temperature of Izmit Bay is around 7 °C. This suggests that there are no physico-chemical barriers in the region to hinder the population spread of *Styela clava*. The Sea of Marmara’s specimens had ripe gonads, indicating its successful reproductive capacity in the area. At this stage it could be concluded that this species is established and has formed a proliferating population in the area.

### The vector for introduction of *Styela clava*

This species has been introduced to different parts of the world’s oceans, including the east Atlantic coast (see Minchin et al. 2006), Australia (Hewitt et al. 1999), New Zealand (Davis and Davis 2006), both coasts of North America (Osman et al. 1989; Lambert and Lambert 1998; Lambert 2003; Wonham and Carlton 2005), Mediterranean Sea (Davis and Davis 2008) and the Black Sea (Micu and Micu 2004). As Davis and Davis (2008) summarized, there are two possible mechanisms of ascidian introduction; shellfish transportation (juvenile ascidians) or via ship’s hulls and sea chests (mature ascidians) (Coutts and Dodgshun 2007). As there is no shellfish farming in the Sea of Marmara, the only possible vector for the introduction of this species to the area was via shipping. The sampling station (K15, Karamursel) is located in İzmit Bay, which is one of the most industrialized areas in Turkey, with intense international ship traffic. The donor area for the Sea of Marmara’s population of this species is unknown at present. It might have been transferred from an area in the Black Sea or/the Mediterranean, or from outside the Mediterranean. Molecular analysis to be performed on the specimens might shed more light on from where this population was originated.

### Impacts of *Styela clava*

The effects of *Styela clava* on soft bottom sediment assemblages in Port Philip Bay were reported to be negligible (Ross et al. 2007). However, Bourque et al. (2007) reported that it caused a decline in mussel production in Canada, as it densely covered mussel lines. It was reported to be an aggressive invader, affecting native fauna by replacing the native competitive dominants in the benthic community (Clarke and Therriault 2007). The economic impact of this species on shellfish production in Canada alone was estimated at between $34–88,000 million (Canadian) per year (Colautti et al. 2006). Experiments made by Osman et al. (1989) indicated that *Styela clava* is capable of greatly reducing the local settlement rate of oysters by preying on their planktonic larvae. The introduction and dense establishment of *Styela clava* in England occurred simultaneously to a sharp decline in the population of the local ascidian, *Ciona intestinalis* (Linnaeus, 1767) (Lützen 1999). *Styela clava* has effectively replaced the indigenous *Pyura haustor* (Stimpson, 1864) and *Ascidia ceratodes* (Huntsman, 1912), which were the dominant ascidian species in southern California (Lambert and Lambert 1998). However, in the Mediterranean Sea, the population level of this species has not increased in the Bassin de Thau in the three years since its discovery in the area and has not affected the shellfish industry greatly. It is thought that summer water temperatures (max. 29.1 °C) and salinity (max. 40.4 psu) in the area might kill off large proportions of the population (Davis and Davis 2009).

## Conclusions

As the Sea of Marmara’s hydrographical conditions conform with the survival requirements of *Styela clava*, it has a great potential to invade the coastal habitats of the Sea of Marmara. In order to stop, or at least mitigate the effects of this invasion, an eradication program should be urgently planned and implemented while the population is still confined to a very small area.
